# Health-Related Quality of Life in Cervical Dystonia Using EQ-5D-5L: A Large Cross-Sectional Study in China

**DOI:** 10.3389/fneur.2022.895272

**Published:** 2022-06-23

**Authors:** Yan Liang, Junyu Lin, Yanbing Hou, Lingyu Zhang, Ruwei Ou, Chunyu Li, Qianqian Wei, Bei Cao, Kuncheng Liu, Zheng Jiang, Tianmi Yang, Jing Yang, Meng Zhang, Simin Kang, Yi Xiao, Qirui Jiang, Jing Yang, Wei Song, Xueping Chen, Bi Zhao, Ying Wu, Huifang Shang

**Affiliations:** Laboratory of Neurodegenerative Disorders, Department of Neurology, Rare Disease Center, West China Hospital, Sichuan University, Chengdu, China

**Keywords:** cervical dystonia, HRQoL, EQ-5D-5L, non-motor symptoms, pain, depression

## Abstract

**Purpose:**

The study aimed to evaluate the health-related quality of life (HRQoL) measured by the five-level EuroQol-5 dimensions (EQ-5D-5L) in patients with cervical dystonia, and to explore the determinants of HRQoL in patients with cervical dystonia.

**Methods:**

EQ-5D-5L health state profiles were converted into a single aggregated “health utility” score. A calibrated visual analog scale (EQ VAS) was used for self-rating of current health status. Multiple linear regression analysis was used to explore the factors associated with HRQoL in cervical dystonia.

**Results:**

A total of 333 patients with cervical dystonia were enrolled in the analysis, with an average age of 44.3 years old. The most common impaired dimension of health was anxiety/depression (73.6%), followed by pain/discomfort (68.2%) and usual activities (48%). The median health utility score was 0.80, and the median EQ VAS score was 70.2. Multivariate linear regression analysis indicated that disease duration and the scores of the Hamilton Depression Rating Scale (HDRS), Pittsburgh sleep quality index (PSQI), Toronto Western Spasmodic Torticollis Rating Scale (TWSTRS) Part I, and TWSTRS Part III were associated with the health utility scores. After adjusting other parameters, the TWSTRS Part III score and the HDRS score were significantly associated with the EQ VAS scores (*p* < 0.05).

**Conclusion:**

This study evaluated HRQoL in patients with cervical dystonia using the Chinese version of the EQ-5D-5L scale. We found that, besides motor symptoms, non-motor symptoms, including depression, pain, and sleep quality, could be greater determinants of HRQoL in patients with cervical dystonia. Management of non-motor symptoms, therefore, may help improve HRQoL in patients with cervical dystonia.

## Introduction

Cervical dystonia (CD) is one of the most common focal dystonias characterized by involuntary contractions of cervical muscles, leading to abnormal movements and posture of head (1). Besides the motor symptoms, non-motor symptoms, such as anxiety, depression, sleep disorders, and pain, are also very common in patients with CD (2). While CD is not a life-threatening disease, it can affect activities of daily living, decrease the quality of life (3), and even cause disability of patients (4).

Most of the previous studies assessed the quality of life in CD using the craniocervical dystonia questionnaire-24 (CDQ 24) or the Short Form-36 Health Survey (SF-36). Additionally, they had usually small sample sizes and yielded inconsistent results. For example, motor severity has been reported to correlate with poor quality of life in CD in some studies (5–7), but not in other studies (8–13).

The five-level EuroQol5-dimensions questionnaire (EQ-5D-5L) is a standardized and more convenient tool to evaluate the health-related quality of life (HRQoL) worldwide (14). It has been extensively used in neurological diseases, such as Parkinson's disease (15), amyotrophic lateral sclerosis (16), and multiple sclerosis (17). The utility values of EQ-5D-5L for Chinese were established in 2017 (18).

Therefore, the aim of this study was to assess HRQoL in patients with CD using the EQ-5D-5L scale in a large Chinese cohort and to explore the determinants of HRQoL in CD.

## Materials and Methods

### Patients Evaluation

We performed a cross-sectional study. All the patients were recruited from the Department of Neurology of West China Hospital of Sichuan University. The patients were diagnosed as CD by neurologists specialized in movement disorders. Only the patients with isolated cervical dystonia were included in the analysis. The patients who had concominant blepharospasm, oromandibular dystonia or dystonia in the limbs or trunk besides CD were excluded in the current study. The study was approved by the Ethics Committee of West China Hospital of Sichuan University (No. 2022-260). All the participants have signed informed consent.

We collected demographic and clinical data of all the participants, including sex, age, age of the onset, and disease duration. All the participants underwent a face-to-face interview by trained movement disorder specialists. Motor and non-motor symptoms were assessed using standard scales. Motor severity was assessed using the Toronto Western Spasmodic Torticollis Rating Scale Part I (TWSTRS-I). Depression was assessed using the Hamilton Depression Rating Scale-24 (HDRS-24) (19). Anxiety was assessed using the Hamilton Anxiety Rating Scale (HARS) (20). Excessive daytime sleepiness was assessed using the Epworth Sleepiness Scale (ESS) scale (21). Sleep quality was assessed using the Pittsburgh sleep quality index (PSQI) scale (22). The global cognitive function was assessed using the Montreal Cognitive Assessment (MoCA) scale (23). The Toronto Western Spasmodic Torticollis Rating Scale Part III (TWSTRS-III) was used to assess pain severity. The Toronto Western Spasmodic Torticollis Rating Scale Part II (TWSTRS-II) was used to assess the activities of daily living.

The HRQoL was assessed using the EQ-5D-5L. EQ-5D-5L comprises two parts. The first part of the EQ-5D-5L assesses five dimensions of health, namely, mobility (MO), self-care (SC), usual activities (UA), pain/discomfort (PD), and anxiety/depression (AD). Each dimension has five levels, namely, no problems, slight problems, moderate problems, severe problems, and extreme problems. The scores of these five problems can be converted into a single aggregated “health utility” score according to the Chinese version of the population-based utility values (18). The second part of the EQ-5D-5L is a self-rating calibrated visual analog scale (EQ VAS), with a range of 0 to 100. Score 0 indicates worst possible health state, while score 100 indicates best possible health state.

### Statistical Analysis

All continuous variables were presented as the mean and standard deviation (SD), and all categorical variables were presented as numbers and percentages. Spearman's correlation analyses were conducted to explore relationships between EQ-5D-5L values (health utility scores and EQ VAS scores) and clinical variables (sex, age, age of the onset, disease duration, scores of TWSTRS-I, TWSTRS-II, TWSTRS-III, HDRS-24, HARS, ESS, PSQI, and MoCA). The multivariate linear regression model was used to explore the factors correlated with the health utility scores and EQ VAS scores of EQ-5D-5L in CD. The health utility scores and EQ VAS scores of EQ-5D-5L were used as dependent variables.

All analyses were performed using the Statistical Package for the Social Sciences (SPSS) version 22.0, and the R. two-tailed *p*-values of < 0.05 were considered statistically significant.

## Results

A total of 333 patients with CD (118 males) were included in the study. The average age of the patients was 44.3 (SD, 13.3) at the baseline, with a mean disease duration of 3.7 (SD, 5.7) years (Table 1).

**Table 1:** Demographic and clinical features of the recruited patients with cervical dystonia.

	**Patients with cervical dystonia**
Total number	333
Male sex, No. (%)	118 (35.4%)
Age, years, mean (SD)	44.3 (13.3)
Age of onset, years, mean (SD)	40.6 (13.3)
Disease duration, mean (SD)	3.7 (5.7)
TWSTRS-I score, mean (SD)	13.4 (5.2)
TWSTRS-II score, mean (SD)	8.2 (6.4)
TWSTRS-III score, mean (SD)	3.1 (3.7)
MoCA score, mean (SD)	25.3 (3.5)
HDRS-24 score, mean (SD)	9.7 (8.0)
HARS score, mean (SD)	8.4 (7.1)
ESS score, mean (SD)	4.0 (4.4)
PSQI score, mean (SD)	6.3 (4.1)
EQ-5D-5L health utility score, mean (SD)	0.8 (0.2)
EQ VAS, mean (SD)	70.2 (15.5)

*TWSTRS-I, Toronto Western Spasmodic Torticollis Rating Scale Part I;TWSTRS-II, Toronto Western Spasmodic Torticollis Rating Scale Part II; TWSTRS-III, Toronto Western Spasmodic Torticollis Rating Scale Part III; MoCA, Montreal Cognitive Assessment; HDRS-24, Hamilton Depression Scale; HARS, Hamilton Anxiety Scale; ESS, Epworth Sleepiness Scale; PSQI, Pittsburgh sleep quality index; EQ-5D-5L, five-level EuroQol 5-dimensions questionnaire; EQ VAS, visual analog scale*.

**Figure 1:**
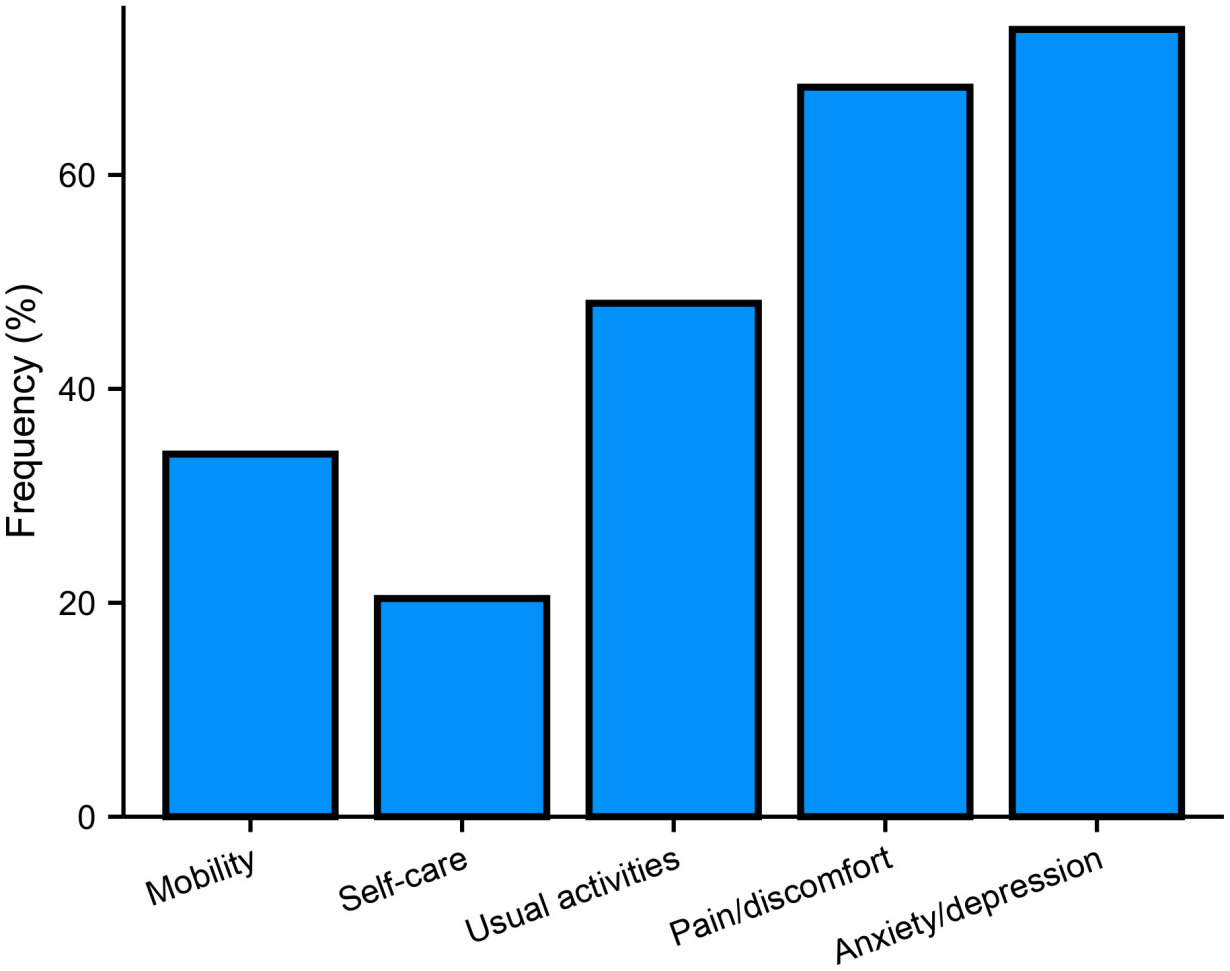
Frequency of reported problems for each of the EQ-5D-5L dimensions.

The median health utility score was 0.80, and the median EQ VAS score was 70.2 for the total patients with CD. Levels 2–5 were considered as impaired for each dimension. The most common impaired dimension of health was anxiety/depression (73.6%), followed by pain/discomfort (68.2%), usual activities (48%), mobility (33.9%), and self-care (20.4%) (Figure 1).

Spearman's correlation analyses showed that the health utility scores were significantly associated with disease duration (*r* = 0.168, *p* = 0.002), the TWSTRS-I score (*r* = −0.314, *p* < 0.001), the TWSTRS-II score (*r* = −0.625, *p* < 0.001), the TWSTRS-III score (*r* = −0.424, *p* < 0.001), the HARS score (*r* = −0.511, *p* < 0.001), the HDRS score (*r* = −0.590, *p* < 0.001), and the PSQI score (*r* = −0.269, *p* < 0.001). The EQ VAS scores were significantly associated with disease duration (*r* = 0.110, *p* = 0.045), the TWSTRS-I score (*r* = −0.141, *p* = 0.010), the TWSTRS-II score (*r* = −0.353, *p* < 0.001), the TWSTRS-III score (*r* = −0.249, *p* < 0.001), the HARS score (*r* = −0.409, *p* < 0.001), the HDRS score (*r* = −0.484, *p* < 0.001), and the PSQI score (*r* = −0.258, *p* < 0.001; Table 2).

**Table 2:** Spearman's correlation analyses of the EQ-5D-5L healthy utility score and the EQ VAS score in patients with cervical dystonia.

	**Healthy utility score**	**EQ VAS**
Sex	0.011	−0.073
Age	0.038	0.029
Age of onset	−0.008	0.025
Disease duration	0.168*	0.110*
TWSTRS-I score	−0.314*	−0.141*
TWSTRS-II score	−0.625*	−0.353*
TWSTRS-III score	−0.424*	−0.249*
MoCA score	0.048	0.042
HDRS-24 score	−0.590*	−0.484*
HARS score	−0.511*	−0.409*
ESS score	−0.007	−0.030
PSQI score	−0.269*	−0.258*

*TWSTRS-I, Toronto Western Spasmodic Torticollis Rating Scale Part I; TWSTRS-II, Toronto Western Spasmodic Torticollis Rating Scale Part II; TWSTRS-III, Toronto Western Spasmodic Torticollis Rating Scale Part III; MoCA, Montreal Cognitive Assessment; HDRS-24, Hamilton Depression Scale; HARS, Hamilton Anxiety Scale; ESS, Epworth Sleepiness Scale; PSQI, Pittsburgh sleep quality index; EQ-5D-5L, five-level EuroQol 5-dimensions questionnaire; EQ VAS, EuroQol visual analog scale.*

* *Significant difference*.

The multivariate linear regression analysis showed that disease duration (β = 0.086, *p* = 0.036) and the scores of the HDRS (β = −0.458, *p* < 0.001), the PSQI (β = −0.094, *p* = 0.035), the TWSTRS Part I (β = −0.216, *p* < 0.001), and the TWSTRS Part III (β = −0.215, *p* < 0.001) were associated with the EQ-5D-5L health utility scores. After adjusting other parameters, the TWSTRS Part III score (β = −0.124, *p* < 0.012) and the HDRS score (β = −0.459, *p* < 0.001) were significantly associated with the EQ VAS scores (*p* < 0.05; Table 3).

**Table 3:** Stepwise linear regression analysis of the total EQ-5D-5L healthy utility score and the total EQ VAS score in patients with cervical dystonia.

	**Variable**	**Standardized**	**SE**	***P*-**
		**regression**		**value**
		**coefficient**		
Healthy utility score	Disease duration	0.086	0.001	0.036*
	HDRS-24 score	−0.458	0.001	<0.001*
	PSQI score	−0.094	0.002	0.035*
	TWSTRS-I score	−0.216	0.001	<0.001*
	TWSTRS-III score	−0.215	0.002	<0.001*
EQ VAS score	HDRS-24 score	−0.459	0.096	<0.001*
	TWSTRS-III score	−0.124	0.204	0.012*

*EQ-5D-5L, five-level EuroQol 5-dimensions questionnaire; EQ VAS, EuroQol visual analog scale; HDRS-24, Hamilton Depression Scale; PSQI, Pittsburgh sleep quality index; TWSTRS-I, Toronto Western Spasmodic Torticollis Rating Scale Part I; TWSTRS-III, Toronto Western Spasmodic Torticollis Rating Scale Part III.*

* *Significant difference*.

## Discussion

The current study describes the HRQoL profile in patients with CD in a large Chinese cohort using the EQ-5D-5L scale. The results showed that anxiety/depression (73.6%) and pain/discomfort (68.2%) were the highest reported dimensions impaired in patients with CD. In addition, multivariate linear regression analysis showed that EQ-5D-5L health utility scores were associated with disease duration, motor severity, and non-motor symptoms, including pain, depression, and sleep quality, while EQ VAS scores were only associated with non-motor symptoms, including pain and depression.

As with our results, non-motor symptoms have been widely reported to play an important role in the decreased quality of life in isolated dystonia, including CD (12, 24, 25). Approximately 55~90% of the patients with CD have been reported to suffer from pain (1, 26, 27). In the current study, pain/discomfort was reported by 68.2% of the patients with CD. Inconsistent with our results, pain has also been identified to affect the quality of life in patients with CD in several studies (7–9, 11–13). The pain in CD can be relieved by botulinum toxin injection (28). However, the mechanism of pain in patients with CD remains largely unknown. The probable mechanisms include both muscle-based and non-muscle-based mechanisms, such as network changes in the basal ganglia (29).

Depression was another determinant of decreased HRQoL in patients with CD identified in the current study. Mood disorders have been reported to be important determinants of poor quality of life in patients with CD in many previous studies (5, 8–11, 13). Depression is common in patients with CD. A recent meta-analysis has yielded depression prevalence of 31.5% in patients with CD (30). In the current study, anxiety/depression was reported by 68.2% of the patients with CD, indicating that the rate of psychiatric comorbidities in CD might be underestimated. Impairment of the dopaminergic system might be an explanation of the development of depression in patients with CD (31).

In line with our results, sleep disorder has also been found to affect the quality of life in patients with CD by a previous study (10). Sleep disorder is also a very common nonmotor symptom in CD (32). Nearly half of the patients with CD have been found to have poor sleep quality (33, 34), which was in accordance with our results (49.8%). Patients with CD with sleep disorders also had a higher pain burden than those with normal sleep (12).

Several studies reported that motor severity was not associated with quality of life in patients with CD (8–13). However, other studies came to the opposite conclusion (5–7). In addition, a study found that motor symptoms had a small influence only on the physical functioning domain of the HRQoL in CD (24). In the current study, motor severity was associated with EQ-5D-5L health utility scores, but not with EQ VAS scores according to the multivariate linear regression analyses. Therefore, the role of motor severity in the HRQoL of CD needs to be validated by more studies in the future.

The results of the current study offered some indications for the strategies of the decreased quality of life in patients with CD. For example, botulinum toxin injection benefits for both pain and motor severity of CD (28), and it has also been reported to help improve the HRQoL in patients with CD (35). In addition, as non-motor symptoms played an important role in the decreased HRQoL in patients with CD, dealing with these non-motor symptoms might be a good choice for improving the HRQoL in CD.

However, several limitations should be acknowledged in the current study. The first limitation was the lack of the healthy controls. The second limitation was that the treatment choices were not included in the analyses.

In conclusion, our study evaluated the HRQoL in patients with CD using the Chinese version of the EQ-5D-5L scale. The results revealed that, besides motor symptoms, non-motor symptoms, including depression, pain, and sleep quality, could be greater determinants of HRQoL in patients with CD, indicating that management of non-motor symptoms may help improve HRQoL in patients with CD.

## Data Availability Statement

The original contributions presented in the study are included in the article/supplementary material, further inquiries can be directed to the corresponding author.

## Ethics Statement

The studies involving human participants were reviewed and approved by Ethics Committee of West China Hospital of Sichuan University. Written informed consent to participate in this study was provided by the participants' legal guardian/next of kin.

## Author Contributions

YL and JL contributed to conception, organization and execution, data collection and statistical analysis, and drafting the manuscript. YH, LZ, CL, QW, BC, KL, ZJ, TY, JY (12th author), MZ, SK, YX, QJ, JY (17th author), WS, XC, BZ, and YW contributed to execution and data collection. RO contributed to conception, organization, execution, and data collection. HS contributed to conception and organization, manuscript review and critique, and was responsible for overall content as the guarantor. All authors contributed to the article and approved the submitted version.

## Funding

This study was supported by the 1.3.5 project for disciplines of excellence–Clinical Research Incubation Project, West China Hospital, Sichuan University (Grant No. 2019HXFH016).

## Conflict of Interest

The authors declare that the research was conducted in the absence of any commercial or financial relationships that could be construed as a potential conflict of interest.

## Publisher's Note

All claims expressed in this article are solely those of the authors and do not necessarily represent those of their affiliated organizations, or those of the publisher, the editors and the reviewers. Any product that may be evaluated in this article, or claim that may be made by its manufacturer, is not guaranteed or endorsed by the publisher.
